# Crosstalk between the Gut Microbiome and Colonic Motility in Chronic Constipation: Potential Mechanisms and Microbiota Modulation

**DOI:** 10.3390/nu14183704

**Published:** 2022-09-08

**Authors:** Ruili Pan, Linlin Wang, Xiaopeng Xu, Ying Chen, Haojue Wang, Gang Wang, Jianxin Zhao, Wei Chen

**Affiliations:** 1 State Key Laboratory of Food Science and Technology, Jiangnan University, Wuxi 214122, China; 2 School of Food Science and Technology, Jiangnan University, Wuxi 214122, China; 3 The Department of Clinical Laboratory, Wuxi Xishan People’s Hospital, Wuxi 214105, China; 4 The Department of of Obstetrics and Gynecology, Wuxi Xishan People’s Hospital, Wuxi 214105, China; 5 National Engineering Research Center for Functional Food, Jiangnan University, Wuxi 214122, China; 6 (Yangzhou) Institute of Food Biotechnology, Jiangnan University, Yangzhou 225004, China

**Keywords:** gut microbiome, microbiota metabolites, constipation, probiotics, 5-hydroxytryptamine, Toll-like receptors

## Abstract

Chronic constipation (CC) is a highly prevalent and burdensome gastrointestinal disorder. Accumulating evidence highlights the link between imbalances in the gut microbiome and constipation. However, the mechanisms by which the microbiome and microbial metabolites affect gut movement remain poorly understood. In this review, we discuss recent studies on the alteration in the gut microbiota in patients with CC and the effectiveness of probiotics in treating gut motility disorder. We highlight the mechanisms that explain how the gut microbiome and its metabolism are linked to gut movement and how intestinal microecological interventions may counteract these changes based on the enteric nervous system, the central nervous system, the immune function, and the ability to modify intestinal secretion and the hormonal milieu. In particular, microbiota-based approaches that modulate the levels of short-chain fatty acids and tryptophan catabolites or that target the 5-hydroxytryptamine and Toll-like receptor pathways may hold therapeutic promise. Finally, we discuss the existing limitations of microecological management in treating constipation and suggest feasible directions for future research.

## 1. Introduction

Primary chronic constipation (CC), including functional constipation (FC) and constipation-predominant irritable bowel syndrome (IBS-C), is characterized by difficult bowel movements and/or a sense of incomplete evacuation, thus influencing quality of life [1]. However, the aetiology and pathophysiology of CC are largely unknown [2]. The epithelial surface of the gastrointestinal (GI) tract is inhabited by a dynamic collection of 40 trillion microbes, referred to as the gut microbiome, which has co-evolved with the host in a mutualistic relationship [3]. Gut microorganisms interact with a host’s metabolism, nervous system, immune system, and endocrine system, impacting its physiological functions [4]. Disruption of the gut microbial communities (dysbiosis) can cause a variety of changes in the host’s pathophysiology that lead to functional gastrointestinal disorders, specifically constipation [5]. Characterization of the microbiome–host crosstalk pathways provides insight into the pathogenesis of constipation.

Recently, the introduction of high-throughput sequencing and metabonomics has provided culture-independent techniques for the exploration of gut microbiota as a whole, rather than on the level of individual microbes. This has contributed to an understanding of the roles that certain microbes and microbial mediators play in constipation [6]. Early reports predominantly pay attention to the relationship between the alteration in intestinal flora and disease states [7]; recent studies have moved beyond associations to define mechanisms of gut microbiota contributing to the constipation-related symptoms [8]. Nevertheless, the precise molecular mechanisms of gut microbiota–host interactions remain to be clarified, especially those relating to the endocrine system. Furthermore, the majority of conclusions are currently derived from animal experiments rather than from studies involving human hosts.

The effective management of constipation remains challenging. The pivotal role of intestinal microbiota in the occurrence and development of constipation has prompted a shift in therapeutic options towards microecological intervention, especially probiotics [9], which has gradually replaced the traditional approaches for treating constipation. However, the mechanisms and effectiveness of probiotics in treating constipation remain to be fully discussed.

In this review, we summarize the existing evidence for the changes in the gut microbiota of children and adults with FC or IBS-C and discuss the mechanisms of microbiota-mediated intestinal motility disorder. Moreover, we outline the evidence for microecological therapy in primary chronic constipation and its possible mechanisms, thereby identifying gaps in existing knowledge and suggesting strategies for improvements in the diagnosis and management of constipation.

A systematic search of published studies was performed using the Google Scholar (https://scholar.google.com; up to 30 May 2022) databases, Web of Science (http://isiknowledge.com; up to 30 May 2022), Cochrane Central Register of Controlled Trials (http://onlinelibrary.wiley.com/cochranelibrary/search; up to 30 May 2022), and Medline (http://www.ncbi.nlm.nih.gov/pubmed; up to 30 May 2022). The search items were diverse because of the large number of specialized terms involved in the mechanism review section, so only some of the search items are listed here for reference. The search terms included “constipation”, “motility”, “gut transit”, “colonic peristalsis”, “microbiota”, “microbiome”, and “metabolites”. These search items were combined with the AND operator to additional search terms for the relevant sections of the review, including “enteric nervous”, “central nervous”, “gut-brain axis”, “immune activation”, “inflammation”, “intestinal barrier”, “faecal water content”, “intestinal secretion”, “progesterone”, “estrogen”, “probiotics”, “symbiotic”, “Bifidobacterium”, “Lactobacillus”, “meta-analysis”, and “randomised controlled trials”.

## 2. Gut Microbiome in Constipation

Intestinal dysbiosis in patients with constipation is mainly characterized by a reduced relative abundance of specific lactate- and butyrate-producing bacteria and an elevated concentration of methanogens, which are potentially pathogenic in children and adults (Table 1) [9]. However, the relevance of these events to the disease state is still controversial. Early research used microbial culture methods to reveal that patients with FC contained significantly lower abundances of *Bifidobacterium* and *Lactobacillus*, while potentially pathogenic bacteria such as *Escherichia coli* and *Staphylococcus aureus* were increased [7]. The emergence of NGS and metagenomic technologies has provided new insights into the role of gut microbiota in gastrointestinal disease. Principal coordinate analysis (PCoA) scores of the 16S rDNA sequencing technique data indicated that the gastrointestinal microbiota composition of constipation is clearly distinct from that of normal individuals. The species diversity of microbiota in the patient samples was lower than that in healthy subjects; it was also accompanied by significantly reduced concentrations of *Bifidobacterium* and *Lactobacillus* and an increased abundance of *Desulfovibrionaceae* [10]. In addition, the study found that the levels of butyrate-producing bacteria, such as *Faecalibacterium* and *Roseburia*, were significantly reduced in patients with FC [10]. It has also been confirmed that the relative abundance of methanogenic bacteria is increased in patients with slow transit constipation relative to healthy subjects [11]. In another study, Yutao Chen et al. collected 3056 fecal amplicon sequence data from five research cohorts and used machine-learning methods to construct the constipation discriminant model. The model identified 15 top-ranking biomarkers, particularly inflammation-related pathogenic bacterial genera *Serratia*, *Dorea*, and *Aeromonas* [12]. Recent advancements have used shotgun metagenomics to identify species within the gut microbiome and have performed functional analysis. Shotgun metagenomics indicated that the relative level of *Roseburia intestinalis*, a prominent butyrate-producing bacterium, was reduced in patients with constipation in comparison to healthy controls, and the microbiome corresponding to constipation was enriched for pathways implicated in methanogenesis [13]. In contrast, the microbiome of healthy individuals was characterized by high levels of genes associated with carbohydrate, fatty acid, and lipid metabolism [14].

However, there is a large amount of conflicting data on the microbial alterations of patients with constipation, relating to changes in the abundances of specific lactate-producing bacteria and *Bacteroides*. A cross-sectional pilot study using 16S rRNA sequencing indicated that there was no change in the abundances of the genera *Bifidobacteria* and *Lactobacilli* in adolescents with obesity and constipation [15] and patients with severe chronic constipation [19]. In elderly patients with CC, levels of *Bacteroides* are significantly increased relative to those in healthy controls [16]. However, Mancabelli et al. found that the abundance of *Bacteroides* in patients with constipation, whose ages spanned the range of 4–94 years, was lower than that in healthy subjects [14]. Notably, de Meij et al. performed IS-pro (a PCR-based microbiota profiling method) to analyze the fecal bacteria of children with constipation and unravelled the highly complex intestinal microbiota composition down to the species; the abundances of the discriminative species of *Bifidobacterium longum*, *Bacteroides fragilis*, and *Bacteroides ovatus* were significantly increased compared with those in healthy children [17]. The conflicting data on the microbial alterations of patients with constipation may not only be attributable to age-related differences and certain individual differences but also to differences in DNA extraction methods (Table 1). The inclusion of a bead-beating step made gram-positive bacterial genera, such as Firmicutes and *Bifidobacterium*, more abundant, resulted in higher microbial diversity, and had a great effect on gut microbiome composition compared to those methods with no mechanical disruption step [20]. Differences in DNA extraction methods might help explain the conflicting findings.

Notably, different intestinal sites harbor certain gut microbiomes, yet the majority of recent research has focused on the analysis of fecal-derived microbiota, which are accessible via non-invasive sampling methods. However, luminal microbiota is generally considered to be representative of the distal large intestinal content. The mucosa-associated microbiota, which live in more intimate contact with the host, cannot be fully replicated by fecal microbiota [21]. In patients with constipation, there is even less similarity between fecal- and mucosa-assocoated microbiota compared with healthy controls and patients with diarrhea [22]. These differences may be due to drier stool allowing fewer signaling molecules to enter the mucosa [23] or the longer transit time providing more opportunities for the communities to diverge [22]. Hence, mucosa-associated microbiota are more likely to affect the host’s epithelial and mucosal function than luminal microbiota [24]. Comparative analyses between fecal and mucosal microbiota showed that the colonic mucosal microbiota composition was correlated with constipation (and was accompanied by a significant increase in *Bacteroidetes*), while the fecal microbial communities were correlated with colonic transit and methane production rather than constipation [18]. However, more evidence is needed to prove the relationship between the mucosal profile and constipation.

In the study above, the dietary intake of the participants was also assessed. Patients with constipation consumed fewer total calories and lower amounts of protein, fat, and fiber, which inevitably affect the gut microbiota and transit. It is unclear whether such dietary habits of patients with constipation are crucial for pathogenesis or whether they represent an adaptive response [25]. In addition, chronic intestinal disease is accompanied by disease exacerbation and remission; a single cross-sectional sampling cannot fully characterize the bacterial composition of the disease which is characterized by temporal heterogeneity [22].

In summary, these findings indicate that patients with constipation appear to have a unique microbiota composition, which differentiates them from healthy individuals. However, due to the large inter-individual differences of the participating patients, the temporal variability of the disease, and the defects of detection methods, the precise differences in the composition of the microbiota are poorly understood. It is necessary to restrict population characteristics, such as age and gender, record dietary habits, and establish standardized methodologies for gut microbiota research. In addition, the identification of microbiota composition down to the strain level to reveal specific microbial profiles, especially for Bifidobacteria and Bacteroidetes, is warranted. Considering that longitudinal sampling overcomes heterogeneity seen in cross-sectional microbiome studies, it is recommended to use longitudinal sampling. Technological advances, particularly capsule endoscopy, have made it possible to investigate bacterial composition along different regions of the intestinal tract. What is more, the relationship between the changes in specific microbial abundance and constipation symptoms warrants further research.

## 3. Potential Mechanisms by Which the Gut Microbiota Modulates Constipation

### 3.1. Gut Microbiota, Enteric Nervous System, and Gut Motility

**Figure 1:**
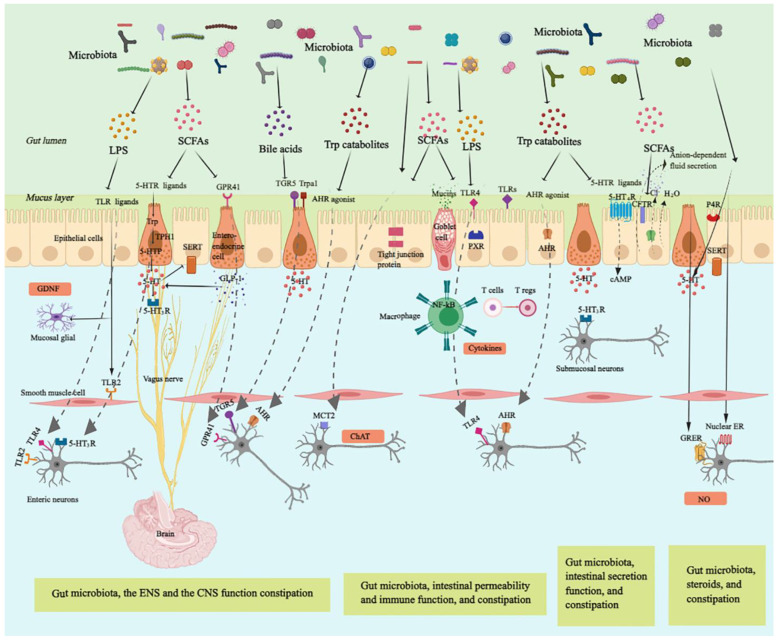
Mechanisms by which the microbiome and metabolites modulate the physiology of constipation. The gut microbiome and its metabolism impact host GI functions via regulation of the ENS, the CNS, the immune system, intestinal secretion, and the hormonal milieu. Notably, microbial-derived SCFAs and indole serve as ligands of 5-HTRs, TLRs, and AHR in the host, thereby facilitating microbiota-mediated functions. For example, the gut microbiome can influence TLR signaling, thereby mediating subsets of enteric neurons and gastrointestinal motility. The metabolite indole activates AHR to maintain mucosal immunity and homeostasis and 5-HT4R to increase colonic fluid secretion and accelerate gastrointestinal transit. Abbreviations: AHR, aryl hydrocarbon receptor; cAMP, cyclic AMP; CFTR, cystic fibrosis transmembrane regulator; ChAT, choline acetyltransferase; CNS, central nervous system; ENS, enteric nervous system; ER, estrogen receptor; GDNF, glial cell-derived neurotrophic factor; GLP-1, glucagon-like peptide-1; GPER, G protein coupled estrogen receptor; LPS, lipopolysaccharide; P4R, progesterone receptors; MCT2, monocarboxylate transporter 2; SCFAs, short-chain fatty acids; SERT, serotonin-selective reuptake transporter; Trp, Tryptophan; TPH1, tryptophan hydroxylase 1; Trpa1, transient receptor potential ankyrin A1; 5-HT, serotonin; TLR, Toll-like receptor; 5-HTP, 5-hydroxytryptophan.

Sensory innervation of the mucosa comes from the enteric nervous system (ENS). The nerve endings are located adjacent to the mucosal sides of absorptive epithelial cells, and thereby are ideally placed to respond to commensal bacteria and modulate intestinal function (Figure 1) [26]. The influence of the gut microbiome on ENS activity is indicated by changes in gut motility patterns (such as the neurogenic colonic migrating motor complexes) [27] and the weakened excitability of enteric neurons in germ-free (GF) mice compared with wild-type mice. These effects lead to a reduction in intestinal transit rates and defects in gut motility [28]. Conversely, the acquisition of a normal gut microbiome can restore the density of the ENS network, increase the excitability of gut sensory neurons [29] and, subsequently, increase gut motility. These effects may be attributed to Toll-like receptor (TLR)-mediated effects of the microbiota on gastrointestinal motility [30]. In addition, gut microbiota also maintains adult ENS, promotes colonic neurogenesis, and regulates colon motility in a TLR2 signaling-dependent manner in mice [31]. Moreover, probiotic and pathogenic bacteria differently regulate TLR2 expression and NO production in human-derived enteric glial cells [32]. Interestingly, the effects of TLR/microbiota pathways on ENS development and homeostasis are mediated by glial cell-derived neurotrophic factor (GDNF) [28,33]. The gut microbiome also contributes to the functional maturation of intestinal neural networks via enteric serotonin networks, thereby initiating the release of 5-hydroxytryptamine (5-HT) by enterochromaffin cells; 5-HT can directly act on the GI tract, causing smooth muscle relaxation or contraction [34]. This observation highlights a potential mechanism that links tryptophan metabolism to functional GI disorders [35]. Thus, microecological management that directly targets specific TLRs and 5-HT signaling pathways may be effective in promoting functional maturation of the ENS and alleviating colonic dysmotility.

Gut microorganisms interact with the ENS not only through bacteria but also through bacterial metabolites and components (Figure 1 and Table 2). Short-chain fatty acids (SCFAs) are the metabolites of fermented dietary fibre and are an important energy source for colonic epithelial cells [36]. SCFAs also function as chemical messengers and signaling molecules that regulate the neurochemical phenotype and functions of the ENS [37]. There are several mechanisms by which SCFAs affect gut motility, though these mechanisms are not fully understood. (1) SCFAs can activate mucosal receptors that are connected to enteric nerves. For instance, SCFAs can stimulate G protein-coupled receptors (GPCRs) (such as GPR41 and GPR43) on enteroendocrine cells (ECs), thereby mediating the secretion of glucagon-like peptide-1 (GLP-1) [38]. (2) SCFAs can modulate 5-HT biosynthesis, thereby influencing colonic peristalsis. For instance, SCFAs can regulate the expression of TpH1 and serotonin-selective reuptake transporter (SERT) in intestinal epithelial cells [39]. (3) Butyrate can regulate the neurochemical phenotype. For example, butyrate enhances the excitability of choline acetyltransferase (ChAT)-positive neurons in a monocarboxylate transporter 2 (MCT2)-dependent manner, thereby improving colonic transit [40]. (4) SCFAs can directly act on colonic and ileal smooth muscle [41].

It should be noted that the regulating effect of SCFAs on gut motility, as mediated by the ENS, may be biphasic [15]. Low concentrations of SCFAs may promote gut motility, while excessive levels may trigger gut dysmotility [42]. Recent studies have emphasized that the dysregulation of tryptophan metabolites plays a key role in the pathogenesis of colonic motility disorders [43,44]. It has been reported that microbiota-generated indole-3-carbinol can activate aryl hydrocarbon receptors (AHRs) within myenteric neurons, enabling them to respond to the microbial environment of the lumen and trigger the expression of neuron-specific effector mechanisms and colonic peristalsis [45]. It has been acknowledged that TLR4 signaling is crucial for enteric neuronal survival and for promoting gastrointestinal motility [46]. Treatment with low-dose lipopolysaccharide (LPS), a microbial cellular component, enhances neuronal survival; however, treatment with high-dose LPS results in neurotoxicity through TLR4 signaling [30].

**Table 2:** Summary of currently known microbial metabolites and their effect on gastrointestinal physiology.

Microbial Metabolites	Effect on Gastrointestinal Physiology	Mechanism	Model Organism
Short-chain fatty acid	ENS function	Stimulation of the ENS receptor type GPCRs to regulate GLP-1 expression [38] Modulation of 5-HT biosynthesis via regulating the expression of TpH1 and SERT [39] Increase in ChAT^+^ neurons to improve colonic transit (Butyrate) [40] Directly acting on the colonic and ileal smooth muscle to stimulate colonic peristalsis [41]	Animal
	CNS function	Stimulation of the mucosal receptors connected to vagal nerves and cholinergic neurons expression [47]	Animal
	Immune activation	Restoring T_regs_ populations and function [48]	Animal
	Intestinal barrier	Activation of AMP-activated protein kinase [49] Stimulating tight junction signaling and the expression of mucin-associated peptides [50] Modulation of goblet cells to release specific mucins, such as MUC2 [51]	Cell
			Animal
			Animal
	Intestinal secretion	Regulation of 5-HT-mediated intestinal fluid and electrolyte secretion via 5-HT_3_R [52] Stimulation of the absorption of water and electrolyte through sodium, water influx, and duodenal bicarbonate secretion [53]	Animal
Tryptophan metabolites	ENS function	Activation of AHR inducing expression of neuron-specific effector mechanisms [45]	Animal
	CNS function	Acting as neuronal modulators to activate Trpa1, which transmit bacterial signals to enteric and vagal nerves (Indole-3-carboxaldehyde) [54]	Animal
	Immune activation	Inducing innate and adaptive immune responses by acting as ligands of AHR [55] Affecting TH17/Treg balance and mucosal homeostasis via IL-22 to attenuate intestinal inflammation in an AHR-dependent manner (Indole) [56]	Animal
	Intestinal barrier	Promotion of barrier integrity by enhancing expression of genes contributing to maintaining the structure and function of epithelial cells (Indole) [57] Enhancement of goblet cell differentiation and mucus secretionn [58] Serving as a ligand for PXR to enhance intestinal barrier [59]	Animal
	Intestinal secretion	Activation of GPCR 5-HT_4_R expressed in the colonic epithelium to elevate amounts of cyclic AMP (cAMP) and anion-dependent fluid secretion [60]	Animal
BAs (especially Chenodeoxycholate and deoxycholate)	ENS function	Activation of TGR5 to release 5-HT and alter gastrointestinal transit [61]	Animal
	Intestinal secretion	Stimulation of colonic secretion through intracellular activation of secretory mechanisms and suppressing of apical Cl^−^/OH^−^ exchange [62]	Cell
Lipopolysaccharide	ENS function	Enhancement of neuronal survival via TLR4 signaling [30]	Animal
	Immune activation	Stimulation of the macrophages to produce pro-inflammatory cytokines via TLR4/ NF-κB pathways [63]	Cell
Surface components of probiotics (surface layer proteins and capsular polysaccharide)	Immune activation	Integration with specific pattern recognition receptors, such as TLRs and NF-κB, to stimulate immune activation [64]	Animal
Methane	ENS function	Acting as the neuromuscular transmitter to impair the neuromuscular function of the gastrointestinal tract to reduce colonic peristalsis [65]	Animal
Hydrogen	ENS function	Enhancement of peristaltic velocity [65]	Animal

Abbreviations: AHR, aryl hydrocarbon receptor; cAMP, cyclic AMP; ChAT, choline acetyltransferase; CNS, central nervous system; ENS, enteric nervous system; GLP-1, glucagon-like peptide-1; GPCRs, G-protein-coupled receptors; IL-22, Interleukin-22; MUC2, mucin 2; PXR, pregnane X receptor; TLR4, Toll-like receptor 4; NF-κB, nuclear factor kappa B; TpH1, tryptophan hydroxylase 1; 5-HT, 5-hydroxytryptamine; 5-HT3R, serotonergic subtype 3 receptor.

Other products of bacterial fermentation [66], such as bile acids and methane, also affect ENS function (Figure 1 and Table 2). Bile acids (BAs) act as signaling molecules to activate G protein-coupled bile acid receptor 1 (GpBAR1, also known as TGR5), which is expressed by EC and myenteric neurons, thereby increasing 5-HT production and accelerating gastrointestinal transit [61]. The equilibrium of hydrogen (H_2_)-methane (CH_4_)-hydrogen sulfide (H_2_S), which are products of microbiota fermentation, play a vital role in the pathogenesis of constipation. The competitive consumption of H_2_ by methanogens and sulfate-reducing bacteria promotes the overproduction of CH_4_ and H_2_S, respectively [67,68]. Excessive methane can serve as a neuromuscular transmitter, regulating the level of serotonin and impairing the neuromuscular function of the GI tract to delay intestinal transit. Additionally, the intestinal environment of a lower water content and higher pH due to slow transit is more conducive to the growth of methanogenic bacteria [69]. Moreover, methane decreased the peristaltic velocity and increased the contraction amplitude of guinea pig ileum in a peristaltic bath, while the opposite phenomenon was detected after hydrogen infusion [65]. A randomized controlled trial (RCT) indicated that a reduction in methane production with antibiotic treatment directed against methanogenic bacteria in the gut might accelerate colonic transit leading to improvement in constipation [70]. However, there are limitations in the use of animal and gas perfusion studies to mimic the physiological status of the intestinal environment, and the links between gas and impaired ENS function need to be further explored.

Collectively, microbiota and microbial mediators, including SCFAs and tryptophan metabolites, appear to modulate gut motility by exerting effects on ENS function and colonic smooth muscle; however, the mechanisms are not fully understood.

### 3.2. Gut Microbiota, the Central Nervous System, and Constipation

Gut microbiota are key regulators of the development, maturity, and activity of the CNS. They can influence the interactions between the gut and CNS through multiple signaling mechanisms, collectively referred to as the bidirectional ‘microbiota-gut-brain axis’ (Figure 1) [71]. Recent advances have revealed the importance of the gut microbiome in modulating brain–gut communication [72], which may participate in the pathophysiology of constipation. Signals to and from the gut and the CNS are dependent on signal transmitters, principally 5-HT and the vagus nerve, which is a key connection within the microbiota–gut–brain axis [73]. Gut microbiota can modulate 5-HT secretion in ECs, while 5-HT receptors (5-HTRs) are highly expressed in vagal afferents [74]. In addition, bacterial metabolites are also potential neuronal modulators (Figure 1 and Table 2). A recent report indicated that indole derivatives produced by Edwardsiella tarda can activate the receptor transient receptor potential ankyrin A1 (Trpa1) on epithelial sensory enteroendocrine cells (EECs). The activation of EECs mediates 5-HT secretion, transmitting bacterial signals to enteric and vagal nerves and leading to increased gut motility [54]. SCFAs also stimulate mucosal receptors connected to vagal nerves, in particular 5-HT3R, which is located on vagal afferent fibres [47]. In summary, these findings support mechanisms by which gastrointestinal microbiota-derived metabolites modulate gut transit by interacting with both the CNS and the gut through the microbiota–EC–vagal afferent. However, the molecular mechanisms by which the microbiota initiates neurotransmitter release, or how the CNS influences the microbiome and its metabolism, thereby influencing the behavior of the brain, are largely unknown.

### 3.3. Gut Microbiota, the Immune System, and Constipation

Seventy percent of the immune system is located in the GI tract. Therefore, the gut is not only a place for digestion and absorption but also the largest immune organ. As such, it is associated with a wide range of diseases, including constipation. Constipation is accompanied by low-level inflammation and damage to the intestinal barrier [7,43]. However, the gut microbiota can regulate the integrity of the epithelial barrier and the mucosal immune system, thereby maintaining intestinal homeostasis [75].

#### 3.3.1. Gut Microbiota, Intestinal Epithelium Barrier Function, and Constipation

The intestinal epithelium, together with the overlying mucus, is mainly constructed from mucins, which provide a physical and immunological barrier against potentially harmful pathogens [76]. It has been suggested that patients with constipation have increased intestinal permeability, as indicated by increased ovalbumin concentrations in serum [7]. This leads to increased exposure to intestinal epithelial bacteria and, subsequently, to the promotion of gut inflammation. A recent study demonstrated that mice colonized with microbiota from patients with constipation had abnormal defecation parameters and decreased MUC2 expression levels. The decreased MUC2 expression levels reduced the release of mucins, suggesting that constipation-induced dysbiosis results in a compromised epithelial barrier [77]. Microbiota can directly mediate the expression of tight junction proteins, such as zonula occludens-1 and claudin-3, or the expression of genes associated with tight junction signaling [78], thus impacting gut immune homeostasis. Microbe-derived butyrate is also capable of facilitating barrier function through several mechanisms, including the activation of AMP-activated protein kinase [49], the stimulation of tight junction signaling, the expression of mucin-associated peptides [50], and the modulation of goblet cells to release specific mucins, such as MUC2 [51]. In addition, tryptophan metabolites (principally indole) have been found to promote barrier integrity by enhancing the expression of genes that contribute to the maintenance of epithelial cell structure and function [57], thereby fortifying goblet cell differentiation and mucus secretion [58]. The symbiotic bacterial metabolite indole propionic acid (IPA) also acts as a ligand for PXR, a physiologic regulator of barrier integrity, thereby protecting intestinal permeability by TLR4 signaling (Figure 1 and Table 2) [59]. However, there is only one human study suggesting that intestinal barrier impairment is involved in the pathogenesis of constipation [7], and more human data are needed to confirm the relationship between constipation and increased intestinal barrier damage.

#### 3.3.2. Gut Microbiota, Immune Activation, and Constipation

Immune activation, including host innate and adaptive immunity, has been found to occur in patients with functional bowel disease [79], and this can be modulated by the gut microbiota (Figure 1). Patients with constipation show an increase in the numbers of CD8+, CD4+, CD3+, and CD25+ T cells and an increase in the proliferation of lymphocytes, revealing the activation of T cell-mediated immunity. Infectious inflammatory-primed CD8+ T cells on the enteric neurons and the flowing immune response led to acute neuronal injury, colorectal distension, and slow colonic transit in mice [7]. Gut microbiota have been found to modulate TLR signaling, thereby influencing the initiation of innate defence responses and intestinal epithelial homeostasis, such as the synthesis of IgA and antimicrobial peptides [80]. In addition, the gut microbiota can modulate the proliferation and differentiation of T cells and induce colonic regulatory T cells (Tregs), which influences the balance of T helper type 17 (Th17) cells and Tregs and immune activation [81], suggesting that Tregs link the gut microbiota to host immune adaptation. Notably, these effects may be attributed to gut microbiota-derived bacterial fermentation products (Figure 1 and Table 2). A report revealed that SCFAs are able to restore the population and function of Tregs in GF mice, modulating inflammation and gut motility [48]. This indicates that metabolites underlie the intestinal adaptive immune response and can improve colonic homeostasis. In addition, accumulating evidence demonstrates that bacterial tryptophan catabolites, which have been described as ligands of AHR (an important factor in the immune response at barrier sites), also induce innate and adaptive immune responses via AHR activation [55]. For example, indole lactic acid was able to affect the differentiation of mouse Th17 cells in vitro [82]. Furthermore, LPS can stimulate the production of pro-inflammatory cytokines (such as tumor necrosis factor (TNF)-α and interleukin (IL)-6) by macrophages via the TLR4/nuclear factor kappa B (TNF-κB) pathway, thereby inducing systemic inflammation [63]. However, the causal relationship between constipation and inflammation, including the role of the gut microbiota, remains poorly understood.

### 3.4. Gut Microbiota, Intestinal Secretion, and Constipation

Normal defecation not only depends on normal gut motility but also on intestinal secretion function. Alterations in the transport of fluid and electrolytes in the intestine represent another pathophysiologic disturbance in constipation [83], which is also affected by gut microbiota (Figure 1). It has been shown that constipation-induced dysbiosis leads to an increase in the water-retaining capacity of the colon and a reduction in the fecal water content [77]. The gut microbiota can regulate the expression of aquaporin; the *Prevotella* (P) enterotype is thought to improve the fecal water content and accelerate gut transit [84]. In addition, microbial mediators regulate the 5-HT-mediated secretion of intestinal fluid and electrolytes (Figure 1 and Table 2). The host’s secretory response to 5-HT might also be mediated by the modulating effects of the gut microbiome on 5-HT_3_R expression within submucosal neurons through acetate production [52]. Tryptamine, a microbiota-generated indole similar to 5-HT, activates the GPCR 5-HT_4_R, which is present on the colonic epithelium, thereby elevating the amount of cyclic AMP (cAMP) and increasing anion-dependent fluid secretion in mice [60]. This results in an increase in the rate of whole-gut transit. In addition, Mars et al. found that tryptamine induced alterations in short-circuit current and promoted fluid secretion in human colonic biopsies [22]. Thus, bacterial metabolites, SCFAs, and tryptophan-derived metabolites can function as ligands of 5-HTRs (5-HT_3_R and 5-HT_4_R, in particular), which are responsible for modulating colonic secretion. BAs (principally chenodeoxycholate and deoxycholate) stimulate colonic secretion through multiple mechanisms, including the intracellular activation of secretory mechanisms, cystic fibrosis transmembrane regulator (CFTR) mediated by cAMP, and the suppressing of apical Cl^−^/OH^−^ exchange [62]. Importantly, recent publications have suggested that intestinal secretion is mediated by microbiota-derived secretory substances, such as SCFAs, tryptamine, and chenodeoxycholate, rather than by inherent defects in epithelial transport of patients with constipation [22].

However, less research has been conducted on intestinal secretion than on intestinal motility, and further study is needed to explore the mechanisms of intestinal secretion mediated by gut microbiota. Furthermore, these results are mainly based on animal models; thus, caution is needed when translating these conclusions to human conditions.

### 3.5. Gut Microbiota, Ovarian Hormones, and Constipation

The overwhelming prevalence of constipation in women may highlight the essential role of sex hormones (such as oestrogen and progesterone) in prolonged stool transit. Clinical and animal studies have shown that hormonal disturbances are linked to the pathogenesis of functional gastrointestinal disorders [85]. In addition, research suggests that the microbiome impacts hormone production and metabolism, and, vice versa, ovarian hormones can influence the proliferation and growth of specific microbes (Figure 1) [86]. Research has demonstrated that a bidirectional relationship exists between the gut microbiota and host sex hormones [87]. Preliminary evidence suggests that the gut microbiota regulates steroid hormone metabolism via steroid enterohepatic circulation [87], bacterial hydroxysteroid dehydrogenase [88], and bacterial genes involved in oestrogen metabolism, such as β-glucuronidase. This regulation leads to oestrogen reabsorption and further alteration in the circulating hormonal profile [89], thereby influencing host physiology [90]. In addition, increased levels of progesterone cause alterations in community structure, characterized by enhanced abundances of *Blautia* and *Bifidobacterium*, and reduced abundances of *Dehalobacterium* and *Bacteroidales* [91], indicating that progesterone contributes to the changes in the gut microbial community during pregnancy [92,93]. Microbial composition is also influenced by estrogen receptor (ER) signaling within colonic mucosa, which modulates intestinal activity and gut motility [94].

Steroid hormones modulate colonic motor functions in a dose-dependent manner through genomic (via nuclear receptors) and/or non-genomic mechanisms (via membrane receptors) [95,96]. These mechanisms are controversial and not completely understood. Recent studies have shown that the stimulation of G protein-coupled oestrogen receptors (GPERs) and nuclear ERs expressed in colonic myenteric neurons may mediate the inhibitory effects of oestrogen on colonic propulsion by promoting nitric oxide (NO) release from myenteric nitrergic nerves [97]. Emerging evidence indicates that the overexpression of nuclear progesterone receptor (P4R), rather than excess progesterone itself, may be responsible for impaired gut motility [98]. These results may explain the 25–40% prevalence of constipation during pregnancy, despite elevated serum progesterone levels in all pregnant women [99]. Overexpression of P4R, which renders muscle cells more sensitive to circulating progesterone, probably leads to impaired colonic contractions (via G-protein signal transduction abnormalities) [100], and an impaired basal motility index involved abnormal concentrations of prostaglandin and COX enzymes [101]. In addition, the overexpression of P4R impairs the contraction of normal muscle cells in response to 5-HT, causing 5-HT signaling abnormalities in a SERT-dependent manner, which may explain the contradictory physiological phenomenon of high levels of 5-HT in patients with constipation [98]. Notably, overexpression of P4R has been found in accessible tissues, such as epithelial cells [98], the circular muscle layer [100], and the outer layer of the muscularis propria [102], suggesting that these abnormalities play a pivotal role in the pathogenesis of constipation. The mechanisms connecting constipation to the overexpression of P4R are largely unknown. Accumulating evidence indicates that the gut microbiota can modulate the expression of receptors and thereby influences disease susceptibility [103,104]. It is reasonable to speculate that perturbations in the microbiota influence the expression of P4R and ER, which results in increased constipation susceptibility; this may be the physiological mechanism of constipation during pregnancy.

Although this field is still in its infancy, limited research shows that hormones and hormone receptors interact with the microbiota, thereby affecting constipation-related symptoms. Notably, the strong connections between hormones, the microbiota, and gut motility provide new and important insights into sex-specific differences between patients with constipation and underscore the importance of considering sex-specific effects in studies on host–microbial interactions. In addition, further exploration of the role of gut microbiota in pregnant women with constipation could reveal the crosstalk of host–hormone–microbiota because of the high incidence of constipation and the dramatic changes in the gut microbiome and hormone levels during pregnancy.

In summary, these findings highlight the importance of the gut microbiome in mediating the ENS, the CNS, the immune system, and intestinal secretion and endocrine functions implicated in constipation. Perturbations in any of these overlapping functions may result in constipation symptoms. Recent advances have also highlighted the importance of microbial metabolites, such as SCFAs and tryptophan-derived compounds, in regulating gut motility and intestinal secretion. Notably, the 5-HT system and TLR signaling have emerged as key players in microbiota-mediated function; exploiting microorganisms that manipulate 5-HT and TLR signaling may pave the way to probiotic-based therapeutic modalities.

## 4. Role of Probiotics in the Treatment of Constipation

Although it is difficult to define the typical microbiota profile of patients with constipation, microecological therapy has attracted significant attention. The potential mechanisms of action of probiotics have also been extensively studied. Probiotics may contribute to the relief of constipation symptoms by modulating the intestinal microenvironment, intestinal epithelial defence responses, and intestinal secretion functions, and by regulating nervous and endocrine systems that influence gut motility and secretion (Figure 1 and Table 3).

### 4.1. Probiotics Relieve Constipation by Modulating the Intestinal Microenvironment

Perturbations in microbiota composition have been implicated in the pathogenesis of constipation. When probiotics dominate the intestinal tract, pathogens have little effect on the host, whereas, if reversed, pathogens can play a key role in intestinal disorders. Probiotic treatment increases the relative abundance of obligate microflora, such as *Bifidobacterium* and *Lactobacillus* spp., and reduces the abundance of potentially pathogenic microflora (Table 3) [127]. Supplementation with *L. casei* strain Shirota considerably increased the abundance of bifidobacteria and lactobacilli [105] in the gut, and treatment with *B. longum* BB536 increased the abundance of bifidobacteria and improved the frequency of defecation [106]. In addition, a study in mice showed that the administration of *Bifidobacterium* spp. improved constipation symptoms, mainly by elevating the ratio of Firmicutes to Bacteroidetes, increasing the abundance of *Lactobacillus*, and reducing the abundance of pathogenic bacterial genera; various species of *Bifidobacterium* (*B. longum*, *B. infantis*, *B. bifidum B. adolescentis*, *B. breve*, and *B. animalis*) displayed species-specific effects on improving constipation [107]. A recent study found that *B. bifidum* and *B. adolescentis* conferred strain-specific effects on the relief of constipation [124,128]; these differing effects may be attributed to the inconsistent effects of probiotics on intestinal flora. Attention should therefore be paid to strain-specific effects during the treatment of constipation. These findings indicate that probiotic supplements may alter the microbial composition of patients with constipation to resemble that of healthy controls and alleviate constipation-related symptoms.

Microbes do not sit idly within the intestine; they must remain metabolically active to survive the environment. Thus, probiotics not only affect the gut microbiota but also affect their fermentation products, especially SCFAs and tryptophan catabolites, which improve gut motility and secretion. Different probiotic species and strains have been proven to improve constipation indicators. Examples include *L. plantarum* IS 10506, which increases SCFA levels [108], and *B. animalis* subsp. *lactis* MN-Gup, which increases the levels of acetate and improves GI transit rates [109]. Emerging data also indicate that tryptophan catabolites produced by tryptophan-utilising species, such as lactobacilli, are key contributors to the maintenance of colon function [110,111].

### 4.2. Probiotics Relieve Constipation by Modulating ENS and CNS Function

Emerging evidence suggests that probiotics alleviate constipation by activating the ENS and the CNS. Probiotics have been shown to prominently modulate enteric neurobiology (Figure 1 and Table 3). Treatment with *Clostridium butyricum* regulates TLR2 expression in enteric neurons, promoting intestinal motility [112]. In addition, the administration of the commensal gut microbe LGG mediates signaling in the ENS via formyl peptide receptor 1 (FPR1), which is expressed on enteric neuronal cells, thereby enhancing the expression of ChAT [113].

It has been shown that probiotics regulate CNS-dependent motility reflexes, hence inevitably sending nerve signals to the brain. For instance, *L. reuteri* enhances the excitability of myenteric neurons in rats and interacts with the gut–brain axis by influencing afferent sensory nerves that regulate bowel movement [114]. *L. rhamnosus* induces mesenteric vagal afferent firing [115], suggesting that probiotics act on the ENS and CNS. In addition, a human study indicated that long-term supplementation with *L. reuteri* DSM-17938 increases gut motility by reducing the levels of 5-HT and brain-derived neurotrophic factor (BDNF) [116], suggesting that probiotics may improve constipation symptoms via the gut–brain axis in humans. However, this needs to be confirmed in further human studies. In summary, the relationship between the microbiota and neural signaling molecules is still not fully clear.

### 4.3. Probiotics Relieve Constipation by Modulating Intestinal Permeability and Immune Function

Probiotics primarily affect intestinal epithelial defence responses by improving the intestinal barrier and innate and adaptive defense responses (Figure 1 and Table 3) [64]. Probiotics can directly augment the expression of tight junction proteins and the MUC2 gene, thereby stimulating mucin secretion by goblet cells and diminishing the binding of intestinal pathogens to mucosal epithelial cells [117]. In addition, it has been shown that probiotic metabolites, such as SCFAs, tryptophan metabolites, and antimicrobial peptides, enhance intestinal epithelial function by competitive adhesion to enterocytes, stimulating SlgA secretion [118], and promoting the expression of genes involved in tight junctions [119]. A recent report showed that butyrate-producing bacteria can alleviate constipation symptoms by increasing the thickness of the mucosal layer [10]. A study by Shi et al. indicated that tryptophan-utilising *L. plantarum* KLDS 1.0386 augments tight junction proteins, elevates mucin mRNA expression and anti-inflammatory cytokine (IL-10) production, and reduces pro-inflammatory cytokine production [119].

The surface components of probiotics, especially surface-layer proteins and capsular polysaccharides, interact with specific pattern recognition receptors, such as TLRs and NF-κB, to mediate immune activation [64]. Probiotics also stimulate adaptive immune responses mediated by intestinal epithelial cells, especially the production of cytokines [129]. For example, B. longum was shown to reduce the secretion of IL-1β and TNF-α in colon tissue, thereby relieving constipation [120]. In addition, indole metabolites of Lactobacillus were shown to affect the TH17 cell/Treg balance [130] and mucosal homeostasis via IL-22, thereby attenuating intestinal inflammation in an AHR-dependent manner [56]. The ability of probiotic bacteria to improve intestinal epithelial defence may be the key mechanism by which probiotics improve intestinal function. However, the effect of probiotics on immunity, and their relationship to gut motility, is largely unknown. Critically, it is unclear whether probiotics relieve constipation, which leads to reduced inflammation, or whether they relieve inflammation, which leads to reduced constipation.

### 4.4. Probiotics Relieve Constipation by Modulating Intestinal Secretion

Probiotics can improve intestinal secretion and accelerate defecation by increasing SCFA levels and modulating 5-HT signaling (Figure 1 and Table 3). *Bifidobacteria* and *Lactobacillus* spp. have been shown to increase the stool water content in mice, which was attributed to elevated SCFA levels [121,128]. Additionally, recent evidence indicates that *B. bifidum* can enhance the expression of 5-HT_4_R, thereby promoting colonic peristalsis and the secretion of intestinal fluid [124]. Furthermore, colonic mucus can act as a lubricant that protects the mucosa and also contributes to stool excretion [131]. Caballerofranco C et al. suggested supplementation with *Lactobacillus plantarum* PS128 to stimulate mucin production [123].

### 4.5. Probiotics Relieve Constipation by Modulating the Function of the Endocrine System

It has been shown that probiotics can regulate sex hormone levels by impacting the structure of gut microbiota (Table 3). Lactobacilli and bifidobacteria have been shown to reduce the estrogen reabsorption rate and adjust the estrogen level by decreasing the relative abundance of β-glucuronidase-producing bacteria [87,132]. *L. plantarum* 30M5 can alter the levels of circulating estrogen by affecting the gut microbiome and its metabolism [125]. In addition, a recent publication showed that probiotic *Enterococcus faecalis* can modulate progesterone levels and Th1-Th2 cell homeostasis [126]. However, research is limited on the effects of probiotics on hormones and hormone receptors (especially progesterone), and, like the immune system, it is currently unclear how probiotics interact with sex hormones to improve constipation.

In summary, it has been shown that probiotics (especially bifidobacteria and lactobacilli) and their metabolites (SCFAs and tryptophan catabolites in particular) exert beneficial effects on constipation, primarily by acting on the luminal environment of the gut, the nervous system, the immune system, and the endocrine system, and by affecting intestinal secretion function. Notably, these regulatory roles are achieved by the microbiome and its metabolic products, which act as ligands for specific host receptors, in particular 5-HTRs, TLRs, and AHR. Therefore, microecological management that targets specific signaling molecules is a promising treatment for constipation. Further research into the microorganisms and mechanisms involved is warranted, especially in terms of the immune and endocrine systems.

## 5. Clinical Applications of Probiotics in the Relief of Constipation

The effects of microecological intervention, especially probiotics, on gastrointestinal symptoms and gut transit time have been studied in an increasing number of randomized controlled trials (RCTs) in children and adults, which are summarized in Table 4.

Bifidobacteria and lactobacilli are the most commonly used types of probiotics for the treatment of constipation. A recent RCT of 103 participants with chronic constipation indicated that the consumption of *B. bifidum* CCFM16 increased the number of weekly spontaneous bowel movements (SBMs) and improved stool consistencies in comparison with a placebo [142]. Similarly, treatment with *B. animalis* subsp. *lactis* GCL2505 increased the frequency of defecation in adults with FC [133]. However, in a RCT that enrolled 159 children with FC, a 3-week administration of *B. lactis* DN-173010 resulted in a considerable increase in stool frequency compared to the baseline, while the increase was comparable in the placebo group, possibly suggesting a placebo effect [134]. In addition, Dimidi et al. reported that the consumption of *B. lactis* NCC2818 (1.5 × 10^10^ CFU/d with continuous intervention for 4 weeks) by patients with mild chronic constipation did not significantly improve the gut transit time, stool frequency, symptoms, or fecal microbiota profiles compared with the placebo group [135]. Furthermore, Ibarra et al. performed a double-blind RCT in which *B. animalis* subsp. *lactis* HN019 was consumed by 228 patients with constipation over a 4-week period and found that there were no statistically significant differences in constipation symptoms after the interventions. However, HN019 was well-tolerated and improved defection frequency in patients with low stool frequency (≤3/week) [136], suggesting that the therapeutic effect of probiotics may depend on the particular symptoms and the severity of constipation. The inconsistent effects of *Bifidobacterium* spp. on constipation may be due to the fact that different probiotic species and strains have different functional and physiological characteristics. Moreover, it is likely that the immunologic, neurologic, and biochemical effects of probiotics are not only strain-specific but also dose-specific.

In the case of Lactobacilli, Koebnick et al. performed the first RCT to investigate the effect of *L. casei* Shirota on CC (in 2003). They found that the probiotic significantly improved gastrointestinal symptoms (with a reported treatment success of 89%), especially defecation frequency [137]. A large RCT of 161 adults with CC demonstrated that patients treated with *L. plantarum* LRCC5193 and *Streptococcus thermophilus* MG510 had better stool consistencies than the placebo group [138]. Notably, after 4 weeks, the level of *L. plantarum* in the stool samples of patients in the treatment group was significantly higher than that of patients in the placebo group, and *L. plantarum* treatment had a lasting effect on stool consistency, even after discontinuation. Another report studied the effect of *L. casei rhamnosus* (Lcr35, 8 × 10^8^ CFU/d for 4 weeks) on 45 children with chronic constipation and demonstrated a remarkable improvement in defecation frequency and a decrease in hard stools in the treatment group compared with the placebo group; Lcr35 was reported to be as effective as MgO in the treatment of children with CC [139]. Nevertheless, another RCT of Lcr35 (8 × 10^8^ CFU twice daily for 4 weeks) in 94 children with functional constipation indicated that the stool frequency of both the placebo and treatment groups significantly increased from baseline to week four, while defecation was more frequent in the placebo group than in the treatment group [140]. In contrast to the previous study, this indicates that Lcr35 was less effective than the placebo in treating children with constipation. These different effects may be due to the sample sizes and differences in the definition of constipation between the two studies.

Some studies have shown that multispecies probiotics are effective in the treatment of constipation. These probiotics have included three (*L. acidophilus*, *B. longum, and S. thermophylus*) [143] or six bacterial species or strains (Duolac Care) [146]. In addition, synergistic effects on the improvement of constipation are seen when multispecies probiotics are combined with prebiotics, combinations that are referred to as synbiotics. For example, it has been shown that the probiotic/prebiotic combinations of Bifid triple viable capsules with soluble dietary fibre [141], *B. coagulans Unique* IS2 with lactulose [145], and a mixture of six types of probiotics with xylooligosaccharide [144] are all effective in synergistic relief of the symptoms of constipation. A recent systematic review and meta-analysis of 15 RCTs involving adults with constipation concluded that the administration of probiotics shortened the gastric transit time by 13.75 h and increased defecation frequency by 0.98 bowel movements/week, while the increase was significant for the treatment with multispecies probiotics, but not *B. lactis* or *B. longum* alone [147]. This result may suggest that multispecies probiotics have a better therapeutic effect than single species on adults with constipation. Because probiotics confer strain- or species-specific effects on constipation symptoms, the advantage of multispecies probiotics may result from synergistic combinations of therapeutic actions.

Notably, recent systematic reviews and meta-analysis indicated that there is no sufficient evidence to support the recommendation of probiotics in the treatment of constipation in children [148,149]. The ESPGHAN and NASPGHAN did not recommend probiotics for the management of childhood constipation [150]. It was suggested that probiotics appeared to be more effective in treating constipation in adults than in children by current RCTs (Table 4) and systematic reviews [147,148,149,150,151]. Probiotics should be used more cautiously in the treatment of constipation in children than in adults.

Currently, international guidelines on probiotic administration for treating gastrointestinal disease remain inconsistent. The American College of Gastroenterology (ACG) clinical guidelines has suggested that the strength of recommendations is conditional. The ACG suggest against probiotics for the management of IBS symptoms [152]. However, the European Society for Primary Care Gastroenterology expert consensus panel recommend that specific probiotics are beneficial in the treatment of certain IBS symptoms and therefore can be used as an adjunct to conventional treatment [153]. The World Gastroenterology Organisation Global Guidelines [154] and International Scientific Association for Probiotics and Prebiotics consensus [155] suggest that specific probiotics exert a pivotal influence on certain lower GI problems, as evidenced by clinical data.

The contradictions in these studies and guidelines may be due to large inter-individual differences, the species- and strain-specific effects of probiotics, sample sizes, symptoms and severity of constipation, and poor methodological quality [9]. The ACG takes into account the lack of rigorous trials based on US FDA endpoints in the current studies evaluating probiotics [152]. Moreover, the composition of patients’ initial gut microbiomes affects the function of probiotics, which may explain the high variability regarding the effects of probiotics on the host [142]. Of note, not all probiotics are systematically tested by preclinical animal experiments, which may be an important reason for the conflicting data.

Given that current investigations did not clearly describe the symptoms and severity of constipation or initial gut microbiota status, it is not clear which patients with constipation will benefit more from probiotic intervention. Although the use of probiotics is generally considered to be safe, extra caution is warranted in children and in individuals with immune dysfunction [156]. Finally, non-viable probiotics have been shown to be effective in improving constipation [157], which suggests that non-live components of probiotics, such as secreted metabolites, may be an alternative microecological therapy for constipation in patients with impaired host defences.

On the other hand, there is some evidence that fecal microbiota transplantation (FMT) might be effective in relieving the gastrointestinal symptoms of patients with constipation and exerts a positive impact on the recovery of gut motility and defecation function by modulating the microbiome [158]. For example, Yan Tian et al. performed FMT on 34 patients with CC and found that FMT treatment promoted intestinal peristalsis and regulated the intestinal microflora [159]. However, only one RCT has been performed yet [160]. More evidence is needed to certify FMT as an available clinical therapy for constipation. Furthermore, FMT is unlikely to be considered as the preferred option due to the challenges in identifying donors as well as the cost and complexity of the procedure. FMT might be more suitable for patients who are refractory to conventional treatment [161].

In summary, there is no clear consensus on the therapeutic effect of probiotics on adults with constipation, and the current limited evidence does not discourage the use of probiotics for the management of childhood constipation. Considering the high heterogeneity of individual studies, the differences in host constipation status, and the strain-specific effects, future RCTs that identify the symptoms and severity of patients with constipation are needed. Given that animal models have a unified genetic background, dietary habits, and disease characteristics, preclinical animal studies are required to preliminary screen effective single- and multi-species probiotics to identify ideal doses, reveal microbiome–host bidirectional interactions, enable more targeted interventions, and fully understand potential synergistic effects. Moreover, the composition of an individual’s initial gut microbiota should be paid attention and determining which gut microbiota are likely to be resistant to colonizing the probiotics used will facilitate clinical applications. What is more, future clinical trials should standardize research methods to facilitate comparison across clinical results.

## 6. Conclusions and Future Perspectives

The gut microbiome plays an important role in the physiology and pathophysiology of constipation. It interacts with the immune system, the ENS, and the CNS, and has the ability to modify intestinal secretion and hormonal milieu. Certain probiotics exert modulatory effects on the host’s physiological processes and can therefore be used to treat constipation. Notably, specific microbial metabolites, such as SCFAs and tryptophan catabolites, and specific signaling pathways, such as TLR-dependent pathways, play central roles in microbiota-mediated intestinal function. Therefore, approaches that manipulate SCFAs and tryptophan catabolites or target 5-HT and TLR pathways are likely to be viable therapeutic strategies for the treatment of constipation. In addition, enteric coating materials that encapsulate microbial metabolites have a significant effect on constipation and are an extraordinarily promising treatment approach. Although these results are encouraging, there are challenges ahead. No consistent “microbial signature” biomarker has been determined for constipation, and evidence for the efficacy of probiotics remains inconsistent due to the high heterogeneity of the individual studies, inconsistent criteria, and strain-specific effects. The precise mechanism of intestinal host–microbial interactions also requires further investigation.

In future research, a rigorous preclinical–clinical trial sequence should be constructed to provide the basis for precise mechanism-based diagnoses and to improve probiotic efficacy for clinical translation. Rigorous identification for the microbial biomarkers is the prerequisite for clinical translation through advanced analyses (such as shotgun metagenomics, metatranscriptomics, metabolomics, and multi-omics methods). Preclinical animal models that can fully simulate different types of constipation, such as humanized gnotobiotic mice, should be used to identify the biological functions of microbial biomarkers and to preliminarily screen probiotics. Further, preclinical investigations can be deployed to identify the physiological characteristics, mechanism of action, dose–effect relationship and potential synergistic effects (multi-strains), and the safety, frequency, and treatment durations of using probiotics. We suggest that future clinical trials should adopt the same criteria and follow trial design and endpoint standards (such as complete spontaneous bowel movements) set out by the European Medicines Agency or US Food and Drug Administration (FDA) [162]. Furthermore, RCTs should utilize large samples, multiple research centers, and uniform definition of constipation (Rome IV), and indicate the symptoms and severity of the disease to identify effective single-strain or microbial formulation for specific types of constipation such as FC or IBS-C. Moreover, the parallel-group, double-blind, and cross-over trials are necessary in clinical trials. A 1- to 2-week screening period and at least an 8-week treatment period followed by a randomized withdrawal design recommended by the FDA may be considered for use in future clinical trials evaluating probiotics. Future trials incorporating the well-acknowledged microbial biomarkers may be more informative to judge the effectiveness of probiotics.

In conclusion, the gut microbiome holds considerable potential as a source of biomarkers for the diagnosis and therapeutic interventions of constipation. Future mechanism-based studies of the gut microbiome will provide insight into next-generation personalized care.

## Figures and Tables

**Table 1:** Reported alteration in gut microbiome in children and adults with constipation.

Study	Subjects	Change	Quantification Method	DNA Extraction Methods
Khalif, I.L., et al., 2005 [7]	57 FC, 25 controls (adults)	*Bifidobacterium* ↓ *Lactobacillus* ↓ *Escherichia coli* ↑ *Staphylococcus aureus* ↑	Microbial culture methods	/
Zhuang, M., et al., 2019 [10]	20 FC, 20 controls (adults)	*Bifidobacterium* ↓ *Lactobacillus* ↓ *Faecalibacterium* ↓ *Roseburia* ↓ *Desulfovibrionaceae* ↑	16S rRNA sequencing (V4)	Cetyltrimethyl Ammonium Bromide (CTAB) method; without bead-beating step
Attaluri, A., et al., 2010 [11]	96 CC, 106 controls (adults)	Methanogenic flora ↑	Breath tests	/
Chen, Y., et al., 2021 [12]	3056 fecal amplicon sequence data from five constipation research cohorts	*Serratia* ↑ *Dorea* ↑ *Aeromonas* ↑	Machine-learning methods	Commercial kits; with or without mechanical disruption step
Tian, H., et al., 2021 [13]	50 FC, 40 controls (adults)	*Roseburia intestinalis* ↓ *Haemophilus* ↓ *parainfluenzae* ↓ *Megamonas unclassified* ↓ *Klebsiella pneumoniae* ↓ *Alistipes putredinis* ↑ *Parabacteroides merdae* ↑ *Odoribacter splanchnicus* ↑ *Eubacterium eligens* ↑	Shotgun metagenomics	QIAamp DNA Stool Mini kit; without additional bead-beating step
Mancabelli, L., et al., 2017 [14]	68 FC, 79 controls (children and adults)	*Bacteroides* ↓ *Roseburia* ↓ *Coprococcus* 3 ↓	16S rRNA sequencing and shotgun metagenomics	QIAamp DNA Stool Mini kit; without additional bead-beating step
Zhu, L., et al., 2014 [15]	8 FC, 14 controls (children)	*Bifidobacteria* ↔ *Lactobacilli* ↔ *Prevotella* ↓ *Firmicutes* ↑	16S rRNA sequencing (V4-V5)	DNeasy Blood and Tissue Kit; with additional bead-beating step
Guo, M., et al., 2020 [16]	61 FC, 48 controls (adults)	*Firmicutes* ↓ *Proteobacteria* ↓ *Bacteroides* ↑ *Prevotella* ↑ *Lactococcus* ↑ *Ruminococcus* ↑ *Butyricimonas* ↑	16S rRNA sequencing (V3-V4)	Fast DNA SPIN extraction kit; with bead-beating step
de Meij, T.G., et al., 2016 [17]	76 FC, 61 controls (children)	*Bifidobacterium longum* ↑ *Bacteroides fragilis* ↑ *Bacteroides ovatus* ↑	IS-pro	Bacterial lysis method; without bead-beating step
Parthasarathy, G., et al., 2015 [18]	25 CC, 25 controls (adults)	*Lactococcus* ↓ *Butyricimonas* ↑	16S rRNA sequencing (V3-V5)	MoBio DNA extraction kit; with bead-beating step
Yarullina, D.R., et al., 2020 [19]	15 CC, 10 controls (adults)	*Roseburia* ↓ *Coprococcus* ↓ *Faecalibacterium* ↓ *Lactobacillus* ↔ *Bifidobacteria* ↔	Culture-based and 16S rRNA sequencing techniques (V3-V4)	Fast DNA SPIN extraction kit; with bead-beating step

↑ Significant increase; ↓ significant decrease; ↔ no change; chronic constipation: CC; functional constipation: FC.

**Table 3:** Summary of currently known probiotics and their role in gastrointestinal physiology.

Probiotics	Effect on Gastrointestinal Physiology	Mechanism	Model Organism
*L. casei strain* Shirota	Modulation microenvironment	Elevation in Bifidobacteria and Lactobacilli abundance [105]	Adults with a stronger tendency to constipation
*B. longum* BB536		Increase in Bifidobacteria abundance to improve the frequency of defecation [106]	Adults with low defecation frequencies
*B. bifidum*		Increase in the ratio of Firmicutes to Bacteroidetes and the amount of Lactobacillus and decrease the levels of pathogenic bacteria [107]	Animal
*L. plantarum* IS 10506		Enhancement of SCFA levels to promote gut motility [108]	Adults with FC
*B. animalis* subsp. *lactis* MN-Gup		Improvment of acetate levels to improve GI transit rate [109]	Animals and adults with FC
*L. gallinarum*		Breaking down tryptophan and modulation of gut microenvironment to improve colon function [110,111]	Animal
*Clostridium butyricum*	ENS and CNS function	Regulation of TLR2 signaling pathway to promote intestinal motility [112]	Animal
*L. rhamnosus GG*		Enhancement of the expression of choline acetyltransferase and gut motility via FPR1 [113]	Animal
*L.reuteri*		Mediation of the excitability of myenteric neurons and interaction with the gut–brain axis by influencing afferent sensory nerves to regulate bowel movement [114]	Animal
*L. rhamnosus*		Modulation of mesenteric vagal afferent firing [115]	Animal
*L.reuteri* DSM-17938		Reduction in 5-HT and BDNF levels to ameliorate constipation [116]	Adults with FC
*L.rhamnosus* (MTCC-5897)	Intestinal permeability and immune function	Augment the expression of tight junction proteins and MUC2 gene to stimulate mucin secretion by goblet cells [117]	Animal
Butyrate-prodution bacteria		Enhancement of mucosal layer to alleviate constipation symptoms [118]	Animal
*L. plantarum* KLDS 1.0386		Augment tight junction proteins amd mucin mRNA expression and anti-inflammatory cytokine (IL-10) levels, and reduction in pro-inflammatory cytokine levels by metabolizing tryptophan [119]	Animal
*B. longum*		Decrease in the concentrations of IL-1β and TNF-α in the colon tissue and increase in the expression of occludin to improve constipation [120]	Animal
*L.plantarum* CQPC02	Intestinal secretion function	Improvement of the water content in stool associated with stimulatory effects of elevated SCFAs on water and electrolyte absorption [121]	Animal
*L.plantarum* LRCC5193		Promotion of intestinal fluid secretion in rats [122]	Animal
*L.plantarum* PS128		Increase in mucin production [123]	Animal
Bifidobacterium (*B.bifidum* and *B.* *animalis* ssp.)		Modulation of 5-HT_4_R expression to promote colonic fluid secretion [124]	Animal
Lactobacilli and bifidobacteria	Hormonal milieu	Decrease in the estrogen reabsorption rate and adjustment of the estrogen level via decreasing the relative abundance of bacteria producing β-glucuronidase [87]	Animal
*L. plantarum* 30M5		Alteration in the levels of circulating estrogen by affecting gut microbiome and its metabolism [125]	Animal
*Enterococcus faecalis*		Modulation of progesterone levels and Th1-Th2 homeostasis [126]	Animal

Abbreviations: BDNF, brain-derived neurotrophic factor; CNS, central nervous system; ENS, enteric nervous system; FPR1, formyl peptide receptor 1; IL-10, Interleukin 10; IL-1β, Interleukin-1β; MUC2, mucin 2; SCFAs, short-chain fatty acids; TLR2, Toll-like receptor 2; TNF-α, tumor necrosis factor-α; 5-HT, 5-hydroxytryptamine; 5-HT4R, serotonergic subtype 4 receptor.

**Table 4:** Randomized controlled and parallel-group trials of micro-ecological intervention for constipated individuals in children and adults.

Study	Population	Probiotic	Intervention	Main Outcome
Ishizuka A, T.K., et al., 2012 [133]	17 adults with FC	*B. animalis* subsp. *lactis* GCL2505	Four consecutive 2-week periods (10^10^ CFU/d)	Supplementation with GCL2505 increased the defecation frequency (+0.5 times/week, *p *< 0.05) and there was no significant change in stool quantity (*p *< 0.1).
Tabbers, M.M., et al., 2011 [134]	159 children with FC	*B. lactis* DN-173 010	Twice a day for 3 weeks (8.5 × 10^9^ CFU/d)	There was no statistically significant change in the stool frequency (4.5 times/week vs. 3.9 times/week, *p* = 0.31) and stool consistency between probiotic group and placebo (mean score of 3.3 vs. 3.5, *p* = 0.07).
Dimidi E, Zdanaviciene A, et al., 2019 [135]	79 adults with FC	*B. lactis* NCC2818	4 weeks (1.5 × 10^10^ CFU/d)	There was no statistically significant change in the gut transit time, stool frequency, stool output, symptoms, stool consistency, or quality of life and *Bifidobacterium* concentrations (*p *< 0.05) between *B. lactis* NCC2818 treatment group and placebo group.
Ibarra, A., et al., 2018 [136]	228 adults with FC	*B. animalis* subsp. *lactis* HN019	4 weeks (1 × 10^9^ or 1 × 10^10^ CFU/d)	There was no statistically significant differences in constipation symptoms after interventions (*p* < 0.05); *B. animalis* subsp. *lactis* HN019 administration improved the BMF in patients with low stool frequency (≤3 times/week) (high dose: +2 times/week, low lose: +1.7 times/week, placebo: +0.8 times/week, *p*= 0.01).
Koebnick, C., et al., 2003 [137]	70 adults with CC	*L. casei* Shirota (LcS)	4 weeks (65 mL/d of a probiotic beverage containing LcS)	Treatment with LcS increased defecation frequency by 3 times/week (*p *= 0.04), increased percentage of treatment success (Lcs: 89%, placebo: 56%, *p *= 0.003), reduced the incidence of severe constipation (Lcs: 34%, placebo: 83%, *p *< 0.001).
Yoon, J.Y., et al., 2018 [138]	171 adults with CC	*Streptococcus thermophilus* MG510 and *L. plantarum* LRCC5193	4 weeks (3.0 × 10^8^ CFU/g *Streptococcus thermophilus* MG510 and 1.0 × 10^8^ CFU/g *L.* *plantarum* LRCC5193)	Probiotics improved stool consistency indicated by the Bristol Stool Form Scale in the probiotic group compared with placebo group (3.7 ± 1.1 vs. 3.1 ± 1.1, *p *= 0.002) and quality of life (*p *= 0.049).
Ling-Nan, B.U., et al., 2007 [139]	45 children with CC	*L. casei rhamnosus* Lcr35	Once daily for 4 weeks (8 × 10^8^ CFU/d)	Administration of *L. casei rhamnosus* Lcr35 significantly increased defecation frequency (0.57 ± 0.17 times/day vs. 0.37 ± 0.1 times/day, *p *= 0.03), reduced the incidence of hard stools (22.4 ± 7.9% vs. 75.5 ± 6.1%, *p *= 0.03), and the percentage of treatment success compared to the placebo group (77.8% vs. 11.1%, *p *= 0.002).
Wojtyniak, K., et al., 2017 [140]	94 children with FC	*L. casei rhamnosus* Lcr35	Twice daily for 4 weeks (1.6 × 10^9^ CFU/d)	The defecation frequency in the placebo group was significantly greater than in the Lcr35 group (+4 times/week vs. +2 times/week, *p *< 0.01).
Chao, D., et al., 2016 [141]	100 adults with FC	Bifid triple	Twice daily for 12 weeks (0.63 g of bifid triple viable capsules and 8 g of soluble dietary fiber)	Synbiotic intake dramatically enhanced clinical remission rates (64.6% vs. 29.2%, *p *< 0.01), reduced colonic transit time (49.3 ± 11.7 vs. 70.5 ± 12.1, *p *= 0.03), improved the stool consistency score (3.5 ± 1.1 vs. 2.4 ± 0.8, *p *< 0.001).
Wang, L., et al., 2022 [142]	103 adults with CC	*B. bifidum* CCFM16	4 weeks (2 × 10^9^ CF U/d)	Treatment of *B. bifidum* CCFM16 increased SBMs (+0.736 SBMs peer week vs. +0.36 SBMs peer week, *p* = 0.116) and obviously improved BSFS (+0.925 vs. +0.2, *p* = 0.0019) compared with placebo.
Tjokronegoro, S.D.P., et al., 2020 [143]	78 children with FC	*L. acidophilus*, *B. longum*, and *S. thermophylus*	Twice a day for 4 weeks (2 × 10^9^ CFU/d)	Probiotics treatment significantly improved stool consistency (27/39 vs. 17/39, *p* = 0.022) and difficulty of defecation (31/39 vs. 20/39, *p* = 0.009) compared with placebo. Overall, relief of constipation with probiotics was better than placebo (31/39 vs. 18/39, *p* = 0.002).
Kim, M.C., et al., 2021 [144]	30 adults with FC	ID-HWS1000 contained six types of probiotics and xylooligosaccharide	4 weeks (one packet a day)	ID-HWS1000 greatly ameliorated the discomfort related to bowel movements, including number of irritable bowel movements compared with placebo (*p* < 0.001).
Venkataraman, R., et al., 2021 [145]	150 adults with FC	*B. coagulans* Unique IS2 and lactulose	4 weeks (*B. coagulans* Unique IS2, 2 × 10^9^ spores) with lactulose (10 g)	There was significant improvement in number of bowel movements in synbiotic groups compared to lactulose or probiotics treatment alone at 3 weeks (*p* < 0.001), while the difference was insignificant at 4 weeks. Probiotics combined with lactulose were significantly more effective and required less time to achieve normal fecal consistency than lactulose (*p* < 0.001).

BMF: bowel movement frequency; SBMs: spontaneous bowel movements; BSFS: Bristol Stool Form Scale.

## Data Availability

Not applicable.
